# Neural Networks of Knowledge: Ontologies Pioneering Precision Medicine in Neurodegenerative Diseases

**DOI:** 10.2174/011570159X353727250314065140

**Published:** 2025-07-14

**Authors:** Pooja Mittal, Rupesh Kumar Gautam, Himanshu Sharma, Rajat Goyal, Garima, Ramit Kapoor, Dileep Kumar, Mohammad Amjad Kamal, Shafiul Haque, Siva Nageswara Rao Gajula

**Affiliations:** 1 GITAM School of Pharmacy, GITAM (Deemed to be) University, Rudraram, Patancheru, Sangareddy Distt, Hyderabad, India;; 2 Department of Pharmacology, Indore Institute of Pharmacy, IIST Campus, Rau, Indore, India;; 3 Chitkara College of Pharmacy, Chitkara University, Rajpura, Punjab, India;; 4 MM College of Pharmacy, Maharishi Markandeshwar (Deemed to be University), Mullana-Ambala, Haryana, 133207, India;; 5 Bristol Myers Squibb, Hyderabad, India;; 6 Department of Pharm Chemistry, Manipal College of Pharmaceutical Sciences, Manipal Academy of Higher Education Manipal, 576104, Karnataka, India;; 7 Department of Entomology and Nematology, UC Davis Comprehensive Cancer Center, University of California Davis, One Shields Avenue, Davis, CA 95616, USA;; 8 Department of Pharmaceutical Sciences, College of Pharmacy, Princess Nourah bint Abdulrahman University, Riyadh, Saudi Arabia;; 9 Department of Pharmacy, Faculty of Allied Health Sciences, Daffodil International University, Dhaka, Bangladesh;; 10 Centre for Global Health Research, Saveetha Medical College and Hospital, Saveetha Institute of Medical and Technical Sciences, Chennai, Tamil Nadu, India;; 11 Department of Health Sciences, Faculty of Science, Novel Global Community Educational Foundation, Hebersham, New South Wales, Australia;; 12 Department of Nursing, College of Nursing and Health Sciences, Jazan University, Jazan, 45142, Saudi Arabia;; 13 School of Medicine, Universidad Espiritu Santo, Samborondon, 091952, Ecuador;; 14 Department of Pharmaceutical Analysis, GITAM School of Pharmacy, GITAM (Deemed to be)University, Rushikonda, Visakhapatnam, Andhra Pradesh, 530045, India

**Keywords:** Neurodegenerative, ontologies, precision medicine, Alzheimer's, Parkinson's, Huntington's

## Abstract

The review focuses on the ways that ontologies are revolutionising precision medicine in their effort to understand neurodegenerative illnesses. Ontologies, which are structured frameworks that outline the relationships between concepts in a certain field, offer a crucial foundation for combining different biological data. Novel insights into the construction of a precision medicine approach to treat neurodegenerative diseases (NDDs) are given by growing advancements in the area of pharmacogenomics. Affected parts of the central nervous system may develop neurological disorders, including Alzheimer's, Parkinson's, autism spectrum, and attention-deficit/hyperactivity disorder. These models allow for standard and helpful data marking, which is needed for cross-disciplinary study and teamwork. With case studies, you can see how ontologies have been used to find biomarkers, understand how sicknesses work, and make models for predicting how drugs will work and how the disease will get worse. For example, problems with data quality, meaning variety, and the need for constant changes to reflect the growing body of scientific knowledge are discussed in this review. It also looks at how semantic data can be mixed with cutting-edge computer methods such as artificial intelligence and machine learning to make brain disease diagnostic and prediction models more exact and accurate. These collaborative networks aim to identify patients at risk, identify patients in the preclinical or early stages of illness, and develop tailored preventative interventions to enhance patient quality of life and prognosis. They also seek to identify new, robust, and effective methods for these patient identification tasks. To this end, the current study has been considered to examine the essential components that may be part of precise and tailored therapy plans used for neurodegenerative illnesses.

## 1 INTRODUCTION

### 1.1 Overview of Neurodegenerative Diseases

Neurodegenerative disorders are classified according to their clinical presentation; extrapyramidal and pyramidal movement abnormalities, together with cognitive or behavioural issues, are the most common forms [1]. The majority of individuals have a combination of clinical characteristics, with very few having pure syndromes. A neuropathological assessment performed at autopsy is currently the gold standard for diagnosis. Neurodegenerative diseases are frequently characterized by anatomic vulnerability and specific protein accumulations. They also have a lot in common, including neuroinflammation, all of which contribute to the gradual malfunction and death of neurons. It is important to keep in mind that there are several neurodegenerative disease processes that can affect an individual and that protein abnormalities common to these diseases can exist before clinical symptoms appear [2]. Generally speaking, diagnostic biomarkers are still lacking, with the possible exception of extremely rare instances in which a genetic mutation causing the illness may be identified. A particular neuropathologic diagnosis in these disorders is primarily made possible by aberrant protein conformations and their distribution in the cellular and neuroanatomic domains. Astrocytic plaques, thorn-shaped astrocytes, and tufted astrocytes are where tau accumulates. While α-synuclein is present in glial cytoplasmic inclusions inside oligodendroglia, tau accumulates in coiled structures [3]. An abnormal structure, similar to that of amyloid, is often present in the protein. Most form filaments have enrichment of β-pleated sheets in their secondary structures. Due to its enhanced interlaboratory and inter-rater reliability, immunohistochemistry is now the preferred approach for studying neurodegenerative disorders and certain other illnesses. This review addresses evidence of functional connectivity from *in vivo* imaging, and the results described herein have ushered in a new paradigm for identifying vulnerability in neurodegenerative disorders.

### 1.2 Precision Medicine Role in Neurodegenerative Disease Management

The burden of neurodegenerative diseases is rising, which raises mortality and morbidity rates as well as healthcare expenses for treatment, hospital stays, and care support. This is one of the main effects of increasingly aging populations [4]. In Europe, 16% of the population is over 65 now; a wide range of age-related neurological illnesses are referred to as neurodegenerative disorders, and they are typified by the gradual loss or malfunctioning of neurons in certain brain and/or spinal cord regions. Individuals afflicted by these conditions exhibit a range of clinical characteristics, such as movement disability, speech difficulty, and cognitive decline.

Dementias are the most common neurodegenerative illnesses; they impact around 7 million individuals in Europe and are expected to triple by 2040 [5]. Patients require specialist care and, when available, medication to manage their limitations over the two to ten years that the disease typically takes to progress. The yearly cost of treating patients with neurodegenerative illnesses is expected to be €130 billion. From this vantage point, national healthcare systems, as well as society at large, are seriously threatened by the rapidly aging population and the growing prevalence of chronic diseases (Alzheimer's disease; AD and Parkinson's disease; PD) are the neurodegenerative disorders most extensively investigated, as indicated by several dedicated databases and research programmes [6].

### 1.3 Importance of Ontologies in Precision Medicine

When biomedical technology advances, the amount of data in the precision medicine (PM) area rises dramatically. The information that has been spread includes important details on the locations of linked biological units and the semantic linkages between them. In order to capture the links between illnesses, phenotypes, genes, mutations, medications, and so on officially and to effectively integrate heterogeneous data, a model for representing information as ontology has to be constructed [7]. Rearranging and characterising medical terminology is the main goal of ontologies' application in the healthcare industry.

In order to communicate and store patient-specific information as well as general medical knowledge effectively, health practitioners have improved their technical terminology and language [8]. With a significant amount of innate information, these terminologies are designed with human processing in mind. A detailed examination of the conception and meanings of these terminologies is necessary to ensure that health information systems convey intricate and all-encompassing medical ideas without any ambiguity [9]. This is a difficult task. On the other hand, it may be accomplished by creating medical domain ontologies to describe these information systems. This review's main goal is to provide an understanding of ontologies and how they are used in computational reasoning to support translational research as well as accurate patient diagnosis and care management.

#### 1.3.1 Data Integration

Transcriptomic, proteomic, and genomic data are examples of the many data sources that may be integrated. These data types require a systematic approach utilising ontologies since they are not easily integrated into integration procedures [10]. Ontologies are used to integrate disparate sources and provide a framework for common understanding, enabling the discovery of patterns and indications for the linkages that would be impractical to find by human reasoning.

#### 1.3.2 Semantic Interoperability

Thus, semantic interoperability becomes a crucial component of precision medicine by operationalising data exchange and interpretation amongst various systems [11]. If not handled in ways that facilitate integration and analysis, data from many sources may become incompatible in this way. By expanding on ideas and connections, ontologies offer a foundation for shared conceptualisation, enabling data to be conceptualised and understood throughout many systems. With the aid of downloadable ontologies, it may interface with repositories in the public domain. It uses specialised ontologies like the MPEG (Moving Picture Expert Group) Ontology and general knowledge-based ones like the Gene Ontology (GO) to enable researchers to work with comparable gene expression data from multiple sources, which simplifies understanding and discovery [12].

#### 1.3.3 Data for Decision-Making

Finding patterns and correlations in data that support clinical judgement and contribute to precision medicine is made feasible by ontologies. Through the process of analysing and contrasting massive datasets, researchers are able to identify biomarkers that may be useful in the prediction of illness risk, build tailored therapies, and create effective treatments [13]. Clinicians will find great assistance in many of the critical choices about patient care from ontologies, which guarantee that such discoveries are the product of methodically gained knowledge about biological processes. Using the Cancer Genome Atlas (TCGA) project as an example, ontologies are essential for the integration and examination of extremely vast datasets in this comprehensive endeavour to characterise the genetic landscape of cancer [14]. Using standardised ontologies like the Gene Ontology and NCI Thesaurus, TCGA discovered new biomarkers and therapeutic targets, opening up extremely intriguing possibilities for more individualised cancer therapy.

Nonetheless, there are still a lot of obstacles in the way of precision medicine's revolution. In order to keep them up to date with new discoveries and technical advancements, they require a significant amount of money and skill, both in creation and maintenance [15]. Machine learning and artificial intelligence are also used in the procedures *via* discoveries and advancements that aid in overcoming their difficulties in order to support the creation and curation of ontologies [16].

## 2 UNDERSTANDING ONTOLOGIES

### 2.1 Definition and Concepts of Ontologies

The philosophical sciences and metaphysics are where the term “ontology” first appeared. Ontology is a branch of philosophy that initially studied existence and being, and its founder was Aristotle. Ontology explains the underlying qualities, connections, and essence of all things [17]. The truth lies at the core of ontology; by its very nature, it is independent of one's background, present consciousness, point of view, or degree of worldly knowledge. The concept of ontology was originally taken by researchers investigating artificial intelligence from philosophers. The definition of ontology that is most frequently used is as follows: An idea is the basis of a corpus of formally articulated knowledge [18]. Since then, information scientists and computer scientists, in general, have become interested in the idea of ontology. The word “ontology” has a new meaning in information and computer science literature that is somewhat connected to its philosophical equivalent. Also, the Meaning of ontology in two different contexts is shown in Fig. (**1**). An ontology is often proposed by a group of scholars who need to exchange knowledge in a certain field. We agree to use the following definitions for terms and concepts: A concept is a thinking unit [18]. A term is a concept's lexical representation. We recommend using “term” in place of “entity” and “concept” in place of “object.” Additionally, we concur that “relationships” between ideas are preferable to “relations” between concepts. Domain knowledge is represented and described using ontologies. Concepts used to characterise domain knowledge and the connections among them make up ontology, which is a common understanding of domain knowledge.

### 2.2 Applications of Biomedical Ontologies

The ontology application examples presented in this section were categorised into groups that corresponded to several primary ontology applications, such as data annotation, terminology mapping, natural language processing, and query improvement [19]. Data annotation in many health care systems, diagnoses are annotated with ICD10 codes. The aforementioned data may be conveniently obtained and employed for a variety of statistical analyses, such as contrasting rates of illness among various countries or socioeconomic strata. An extensive body of research examined the relationships among morbidity, mortality, and several population-influencing factors, including stress, air pollution, and other variables. Additionally, the control over healthcare services was supported by ICD10-annotated data. Several kinds of biological and clinical data are annotated using UMLS. Research pertaining to drugs uses NDF-RT. For instance, PharmGKB data entries, including drug-disease relationships, were annotated using it [20]. In order to standardize labelling and facilitate the interchange of information about drug products, it was also suggested as a source vocabulary.

In research conducted, it was effectively employed to look for signs of medication intolerance. Protein sequences and structures are the main annotation tasks for GO. A method to describe gene groups obtained from microarray analysis is described. The so-called GO enrichment analysis techniques are well-reviewed. Studies looking into the links between protein sequence, structure, and function have also made use of GO annotations [21]. In our study, a program for evaluating the quality of protein models was implemented using protein Mapping Terminologies.

## 3 NEURODEGENERATIVE DISEASES: CHALLENGES AND OPPORTUNITIES

Millions of people suffer from a range of severe disorders together, referred to as neurodegenerative diseases, which significantly affect cognition, motor function, and behaviour [22]. The disorders known as neurodegenerative diseases are caused by age-dependent degeneration and death of neurons. Neurodegenerative illnesses are characterised by a gradual loss of neurons from specific parts of the brain and/or spinal cord. Neuronal degeneration can result in loss of motor function, cognitive impairment, and difficulty making decisions [23]. The lack of adequate therapy for these disorders stems from incomplete investigation of the processes underlying their pathophysiologies. It is vital to discover a cure for neurodegenerative illnesses that largely impact motor functions, as the World Health Organisation (WHO) has revealed that these disorders may be the second most prevalent cause of mortality after cardiovascular diseases. More people are affected financially, and there is a greater danger to the public's health when it comes to the most prevalent neurodegenerative illnesses, which include ALS, PD, AD, and Huntington's disease (HD) [24]. Neural degeneration is influenced by hereditary factors as well, even though aggregation and deposition of toxic proteins in the brain, along with malfunctioning mitochondria, are major causes of the disease's course.

The annual cost of treating people with neurodegenerative disorders is around €130 billion. Thus, it is possible to thoroughly examine neurodegenerative illnesses in order to obtain a comprehensive picture and to develop treatment plans that will enable patients who are struggling to receive better care. Based on research programmes and databases like MDSgene, PDGene, JPND research programme, and AlzGene, AD and PD are now the two most studied disorders [25]. Therefore, it is important to build collaborative networks between research institutes, hospitals, and knowledgeable specialists in order to conduct bench-to-bedside research that can improve patient outcomes by identifying diseases early, developing safer therapies, and improving prognoses.

### 3.1 Alzheimer's, Parkinson's, Huntington's, and Other Common Neurodegenerative Diseases

Millions of individuals worldwide are impacted by neurodegenerative diseases. All non-communicable diseases (NDs) are primarily caused by aging. However, new research shows that genetic predisposition and environmental variables can raise an individual's risk for NDs with equal effect [26]. Furthermore, the rate and degree of neurodegeneration are mostly determined by the individual's immediate environment, even though the expression of certain genes responsible for NDs is expressed within the individual. Multiple diseases may highlight a single neurodegenerative condition, according to more recent research.

Thus, the severity and prognosis of NDs vary greatly, with some cases potentially posing a serious risk to life. The brain controls a wide range of physiological processes. In some cases, therapies aim to ameliorate symptoms, relieve pain if it is present, and/or restore movement and balance. Most NDs develop without remission [27]. Analysis of the clinical, neuropathological, and pharmacological dimensions of HD, PD, and AD is shown in Table **1**. We shall touch on a few common NDs in the following sections.

#### 3.1.1 Alzheimer’s Disease (AD)

The development of AD is dependent on tau and amyloid-beta (Aβ) protein accumulation, according to recent studies on the disease's pathophysiology [28]. Plaque growth disrupts the hippocampal circuitry, leading to poor consolidation of short-term memory into long-term traces. The characteristics of AD include extensive neuronal loss, abrupt synaptic connections, and disruption to the essential neurotransmitter systems needed for memory and other brain functions [29]. In the early stages of AD, selective memory impairment is consequently the most common clinical symptom. Additionally, there is frequent damage to functions that are dependent on the hippocampus and medial temporal lobe, such as declarative episodic memory [30]. Ultimately, deficiencies in executive function, judgement, and problem-solving abilities are further clinical indicators that frequently manifest early.

#### 3.1.2 Parkinson’s Disease (PD)

A tremor, muscular rigidity, uneven gait, and issues with balance and coordination are some of the symptoms of PD, a degenerative neurological illness. The PD may also be caused by non-genetic factors [31]. The majority of PD risk factors are thought to be age-related. Several additional variables are also known to influence the chance of developing PD, even if the precise mechanism behind these changes in risk is yet unclear [32]. These include consuming an excessive amount of coffee, smoking, and being near contaminated environments. Parkinson's disease is mostly caused by atrophy of the frontal brain and enlargement of the ventricles [33]. It is the most prominent morphological alteration observed in brains affected by PD, which is characterised by primary cell death that impairs the nigrostriatal pathway [34]. Many processes have been shown to have a crucial role in the development of Parkinson's disease.

#### 3.1.3 Huntington’s Disease (HD)

One prevalent neurodegenerative condition is HD [35]. The neurobiological predictors of Huntington's Disease study has gathered information on over 1300 individuals who are at risk for HD and show early signs of sickness. HD is a neurological disorder that is autosomal dominant. Although the condition often starts in the fourth or fifth decade of life and lasts for 15 to 20 years, it may start at any moment, from early infancy to very old age. Due to the fact that most early-onset patients acquire the mutation from their father HD exhibits an anticipatory inheritance pattern when inherited *via* the male line [35]. This illness often manifests at forty years of age, and in the West, it affects 4–10 persons per 100,000 as the number of CAG repeats rises, glutamine residues near the amino terminus of proteins, known as poly-glutamine (poly Q), elongate [36]. According to Williams and Paulson (2008), this results in aggregation and toxicity. Mutant huntingtin (mHtt), which may assemble and clump in the cytoplasm and nucleus, is the primary characteristic of HD. The most common amyloid fibre components in the mHtt are βSheet configurations. The aggregation protein mHtt's toxicity and insolubility are the main reasons why neurons die in HD [37].

### 3.2 Challenges in Diagnosis, Treatment and Disease Management

Mutant huntingtin (mHtt), an aggregated protein, is a characteristic of HD, one of the most common neuro-degenerative illnesses. The 5′ terminus of the HTT gene contains 7-35 CAG repeats that encode glutamine [42]. Cognitive decline, mental instability, and increasing motor dysfunction in HD are all influenced by neuronal loss and malfunction in the basal ganglia. Furthermore, neurode-generation has also been seen in the thalamus, substantia nigra, cerebellum, globus pallidus, cerebral cortex, subthalamic nucleus, and nucleus accumbens. In the West, this condition is prevalent at a rate of 4-6 cases per 100,000 people, with a mean onset age of 40 years. Aggregation and toxicity are caused by the elongation of glutamine residues, or polyglutamine (polyQ), at the amino terminus of proteins caused by an increase in CAG repeats [43]. The principal player in the HD is mutant huntingtin (mHtt), which may collect and form inclusion forms in the cytoplasm and nucleus. Many different biological processes, including apoptosis and cell death, are influenced by these interactions. Cell death and apoptosis are caused by a variety of signaling pathways that are activated by any disturbance in the hemostasis and dynamic of mitochondria [44].

Neurologists recognised non-PD neuropathologies in 10% of patients receiving treatment for Parkinson's disease, despite the use of stringent clinical diagnostic criteria. Essential tremors and other types of secondary parkinsonism are among the conditions that are frequently misinterpreted as non-PD tremor syndromes. Even for experts in movement disorders, distinguishing between early PD and atypical parkinsonism, a term used in the literature to refer to a variety of neurodegenerative conditions in which there is a difference between PD and some sort of parkinsonian syndrome, is the most difficult diagnosis to make. It can be quite challenging to distinguish between these diseases and PD at a clinically early stage. In clinicopathological investigations, the error rates have been reported to range from 7% to 35%.

Currently, nevertheless, scientists and medical pro-fessionals are exploring whole new avenues for the successful treatment of neurological illnesses. This might entail personalised medicine, wherein therapeutic approaches are categorised according to a patient's unique attributes, such as genetic composition, medical background, and clinical manifestation. Testing genetic variants of certain genes linked to neurological illnesses, such as HD, is one way to target therapeutic approaches [45].

### 3.3 Opportunities for Precision Medicine in the Detection of Neurodegenerative Disease

Though the pathophysiology of many presentations is still unknown, recent biomedical research advancements and innovations have reached extremely advanced levels [22, 46]. Thus, with various features of these illnesses, it appears that no one therapeutic approach can be effective in any such scenario. Neurodegenerative diseases, like Alzheimer's and Parkinson's, are two notable instances of such complex illnesses. When a comparable diagnosis is established, patients with heterogeneous patient populations require uniform therapy [47]. Crucially, distinct patient subgroups have distinct molecular profiles; as a result, identification and categorization into different variations are rather involved and need a great deal of coordinated effort between physicians and researchers. Biomarkers are indeed required that can precisely forecast the clinical outcome of a subtype diagnosis made by a clinician. These biomarkers will enable accurate categorization of the particular variation impacting an individual and, consequently, customised treatment plans based on this data [48].

Currently, the search for biomarkers for neurodegenerative diseases is a popular topic. Quantifiable and objectively assessed indicators are what biomarkers are by definition [49]. They stand for both healthy and unhealthy biological processes as well as pharmacological responses to medical treatments. Enhancing clinical diagnosis or increasing the accuracy of differential diagnosis is the primary goal of biomarkers. Along with therapeutic response rates, biomarkers should aid in determining the stage and rate of a disease's progression. Numerous variables influence the sensitivity and specificity of the biomarkers. The ability of a test to detect circumstances in a person is known as sensitivity. The ability to pinpoint specific situations when the traits being studied do not manifest is known as specificity. The development and progression of neurodegenerative disorders are significantly influenced by both genetic and non-genetic variables, such as lifestyle choices, environmental exposures, microbiome makeup, and co-occurring medical illnesses, in addition to genetic factors. The pathophysiology of these complicated illnesses has been better understood because of technological innovations that have lately revealed thousands of susceptibility variables [50].

### 3.4 The Conceptual Distinction between Customized and Precision Medicine

Personalized medicine involves treating patients based on their genetic composition as well as their choices, beliefs, attitudes, knowledge, and social context, while precision medicine focuses on providing healthcare *via* the utilization of data, analytics, and information. This technique has wide-ranging consequences for our nation's research agenda, as well as for its usage and acceptance in the medical sector beyond the realm of genetics. Precision medicine and its ecosystem rely on digital health, patient-centeredness and engagement, genomics and other molecular technologies, data sharing, and data science [51].

### 3.5 Opportunities for Precision Medicine in Neurodegenerative Diseases Treatment

Compared to the conventional “one drug fits all” strategy, precision medicine therapy and prevention of neuro-degenerative illnesses. Actually, neurodegenerative disorders can exhibit a wide range of clinical manifestations even in individuals with the same illness, making it extremely improbable that a single medication will be beneficial [52]. In the earlier sections of the review, it was discussed how the vulnerability to neurodegenerative illnesses is influenced by genes, neuroepigenetic modifiers, non-genetic variables, and medications [25, 53]. Table **2** shows examples of conditions in which precision medicine has been used, while Table **3** shows the biomarker list, which is used in neurodegenerative diseases. The dynamics of neuro epigenetic alterations that take place inside and between individuals should receive a great deal of attention. By combining all of the data, “omic” profiles and a 360-degree picture of the patient may be produced. Precision medicine may use the resulting “omic” profiles to develop stratified medicine that can classify patients into different therapy groups [54]. Social networks' capacity to facilitate the simultaneous exchange of massive volumes of data globally has made it possible to close the gap caused by physical remoteness and challenges in obtaining these kinds of details.

## 4 ONTOLOGIES IN NEURODEGENERATIVE DISEASE RESEARCH

The ontology for the representation of brain illness data, or NDDO (Neurodegenerative Disease Data Ontology), is the subject of our article. Hospitals gather data on patients' illness progression and neurological diagnostic data (clinical, imaging, biomarker, *etc*. [69]); this ontology makes it easier to annotate datasets semantically. To identify appropriate algorithms for data analytics, text mining, and reasoning were performed across dispersed data and knowledge sources. We expanded and repurposed our earlier work on the ontology of core data mining entities (OntoDM-core) and ontology of data types (OntoDT) to describe particular domain datatypes that appear in the domain datasets to meet the data analytics viewpoint [70]. We present two use cases to illustrate the usefulness of Neurodegenerative Disease Data Ontology (NDDO): enriching clinical process information into neurodegeneration data and semantic annotation of datasets.

Many tools have been developed to study NDs; these can be freely accessible or commercially available. Some Neuroinformatics tools are meant for the analysis, modeling, and visualization of neural systems such as Cytoscape, Gephi, and EEGNET. *In silico* tools for the study of NDs are summarized in Table **4**. Further, the commonly used tools are discussed below [70-73].

### 4.1 Ontology role in Integration of Data and Knowledge Representation

For knowledge search, ontologies organize disorganized data and provide a common framework for decision-making. They enable data integration, facilitate research process management, and enhance computer phenotyping for research investigations [72]. Ontologies are widely used in data-intensive fields for information integration. When merging various types of data, ontological models guarantee unity. Ontologies describe objects and relationships within a field essential for automatically combining knowledge with health data. Ontologists facilitate the automated integration of data from many sources [73]. They make information modeling possible for efficient processing and searching. Ontologies integrate many models with data sources. In integration processes, ontologies represent components and coordination, enable the networking of IoT semantic data merging, and allow for consistent and widespread management of IoT systems. Ontologies classify data into knowledge and information domains [74].

### 4.2 Tools and Resources Based on Ontology for Neurodegenerative Research

Several ontology-based tools and resources have been developed to promote data management, integration, and analysis for neurodegenerative disease research called The NeuroLOG Initiative. This made it possible to integrate a variety of data sets after the fact, giving rise to the ability characterizations needed for any thorough examination of cognitive deficits in neurodegenerative disease [75]. DemKG Framework is an open-source framework that facilitates the creation of knowledge graphs that integrate data from dementia research. By modifying current ontologies and community standards, DemKG integrates several data modalities, like as imaging, deep phenotyping, and multi-omics analysis. This framework will help researchers better understand the causes of dementia and demystify how we can target new treatments by supporting sophisticated data mining and integration [76]. There are more than 2 billion predicted and validated protein interactions in the open-source web-based database STRING. Using a confidence score, users may uncover links between interacting proteins or genes in the STRING database, which consists of protein-protein interaction networks based on functional linkages. PPI of the STRING database is based on the selection of scientifically proven interactions from the literature. Users may use a Uniprot ID, one or more protein names, or one or more amino acid sequences to search the database for a gene or protein. Proteins or genes are represented by nodes, and the detection technique used to build relationships is illustrated by edges of various colours; predicted functional correlations are assigned a confidence level. STRING has various applications in the study of NDs, including establishing PPI networks, assessing the degree of confidence or evidence for each interaction, investigating Reactome pathways, and undertaking gene ontology research.

### 4.3 Case Studies: Applications of Ontologies in Understanding Disease Mechanisms and Drug Discovery

Semantic technologies, such as frameworks, allow the link of information for drug creation insights. The paper rates 34 important ideas in addition to talking about good conceptual uses in medicine-making [77]. Two models that allow trait naming and enable machine-readable descriptions are TrOn and OBA. Ontologies, by giving a full picture of data, help in the finding of causing agents of diseases including Alzheimer's, lupus, and cancer, hence allowing the production of more targeted drugs [78]. Ontologies like BAO, LIFEo, and DTO, which link genes, proteins, and tiny chemicals related to sickness, allow knowledge-based data integration that helps in the finding of new drugs [79].

## 5 PRECISION MEDICINE APPROACHES: GENOMICS, PROTEOMICS, AND METABOLOMICS IN PRECISION MEDICINE

A US National Research Council paper that aimed to stimulate a new taxonomy for illness categorization *via* a knowledge network first popularised the phrase “precision medicine” [80]. In the publication's appendix, the authors elucidate that the term's origin, in contrast to the more widely used term “personalized medicine”, was meant to communicate the idea that while therapeutics were rarely created for individual patients, patient subgroups could be identified, frequently through genomics, and targeted more precisely [81]. Following the State of the Union speech, there was a sharp spike in global internet searches for the word, which has subsequently stabilized at levels comparable to those of “personalized medicine”. More accuracy is also necessary for precision medicine to thrive. Since decreased accuracy results in missed opportunities for discovery, the current genome analysis methods were created with population or cohort variant detection in mind. On the other hand, for people and families affected by hereditary illness, an erroneous clinical genetic test might have extremely catastrophic repercussions [82]. In order to elevate genomics to the level of clinical practice, our community must overcome obstacles in the areas of sequencing technology, algorithm development, and data sharing, which we address after describing the exciting potential uses of precision medicine as it presently stands. Panomic data will be used in the future of medicine to develop more potent diagnostic tools and increasingly individualized treatments based on a patient's genetic uniqueness [83].

### 5.1 Patient Stratification and Personalized Treatment Strategies

In order to make therapeutic techniques more precise, that is, to provide “tailored” or “targeted” therapy, it is necessary to evaluate the factors that make up a novel taxonomy in terms of how well they predict treatment outcomes [84]. Two approaches might direct the process of creating models that forecast treatment outcomes. First, it is possible to use the prognostic and diagnostic models from the earlier phase; in fact, prognostic factors are frequently taken for granted as the obvious variables to take into account in this situation [85]. Another option is to use the data collected directly to apply biological and other expertise, such as in the assessment of mepolizumab, a monoclonal antibody that targets IL-5, as a treatment for asthma. Mepolizumab was reported to be significantly related to a decrease in blood and sputum eosinophils in the initial clinical trials, although it did not seem to significantly improve clinical outcomes for asthma patients [86]. Mepolizumab treatment had notable clinical benefits in this subgroup, lowering exacerbations and raising asthma quality of life ratings. Benralizumab, a monoclonal antibody that targets the IL-5 receptor, has led to an expansion of these findings recently. Once more, asthmatic patients with increased eosinophil counts saw substantially fewer flare-ups [87]. The latter example demonstrates that evaluating the effects of differential treatments frequently entails looking at clinical trial subgroups to see if treatment effects vary significantly across patient groups. Although the concept is simple, there are methodological issues to consider, such as defining clinically meaningful differences in effects and statistical power.

### 5.2 Role of Ontologies in Precision Medicine Workflows

With the advancement of knowledge engineering to the Semantic Web, ontologies are no more static artefacts produced in a closed context by a single research group but rather dynamic products of collaborative development [88]. Nevertheless, community members' participation options, the variety of roles they might play, and the processes they use to negotiate and reach a consensus change during the projects, sometimes significantly. Our goal is to develop a flexible system that can be used in the Protégé scenario to facilitate different types of cooperative procedures. In this paper, we describe the characteristics of the procedures and evaluate them for many active projects [89]. We discuss an ontology we developed to capture several steps in the process of building collaborative ontologies. An integral component of Protégé's adjustable workflow support is this ontology. By formalising DILIGENT and BiomedGT, two unique collaborative techniques that have been reported in the literature, we evaluate the breadth and flexibility of this ontology. This evaluation demonstrates that our built workflow ontology is sufficiently flexible to represent these various actions.

## 6 ADVANCEMENTS AND INNOVATIONS

Ontology-based developments in precision medicine are crucial due to the gaps in our understanding of neurodegenerative illnesses and the need for customised therapy [90]. The current clinicopathologic paradigm, which relies on protein aggregation, has proven ineffective in altering sickness, underscoring the necessity of switching to a mechanism-based disease classification system [91]. Precision medicine in neurodegeneration must address problems related to disease heterogeneity, which necessitates an integrated approach integrating neurology, neuroscience, and psychiatry in order to develop biomarker-guided therapeutics at a systems level. Precision medicine, which would enable tailored therapy based on the needs and sickness symptoms of individual patients, requires a more precise understanding of the molecular processes underlying neurodegenerative illnesses [92].

### 6.1 Emerging Technologies and Techniques in Neurodegenerative Disease Research

Neuroimaging methods like MRI and PET have a major impact on the diagnosis and pathophysiology of diseases like Parkinson's and Alzheimer's [93]. Together with the growing ageing population, one of the biggest concerns facing healthcare systems is the rising prevalence of neurological diseases. Understanding the effects of genes, epigenetic changes, ageing, diet, medications, and microbiota on health and illness has improved greatly as a result of the tremendous advancements in biomedical research and informatics [94]. Regarding this matter, bringing may depend on the establishment of cooperative networks between academic institutions, medical centres, and highly skilled specialists [51]. To this end, the primary elements that might comprise targeted and individualised treatment plans for neurodegenerative diseases have been considered for this study. According to this viewpoint, the development of web-based networks enables the application of precision medicine techniques across various specialised centres, assisting in clinical and therapeutic decision-making, and encouraging the practice of neurodegenerative disease prevention and participation [95].

### 6.2 AI and Machine Learning Applications in Precision Medicine

Precision medicine's progress in healthcare has overtaken the traditional symptom-driven treatment approach by enabling earlier illness risk prediction through enhanced diagnostics and tailored, more efficient therapies [96]. A better understanding of biological markers that might indicate changes in health is the consequence of carefully examining both wide and general patient data in order to watch and distinguish between sick and reasonably healthy individuals and choose the best course towards precision medicine. The integration of artificial intelligence with precision and genomic medicine holds promise for enhancing patient care. Genomic medicine technologies are being used by patients with less common treatment responses or with specific healthcare needs.

### 6.3 Future Directions and Opportunities for Ontology-Based Precision Medicine in Neurodegenerative Diseases

Precision medicine is critical to effective disease prevention and treatment across a range of populations. In many progressive neurodegenerative illnesses, including age-related macular degeneration (AMD), Alzheimer's disease, and glaucoma, multiple physiological systems malfunction [97]. Diseases of the heart, digestive tract, endocrine system, immunological system, and brain system may all result from abnormalities in these systems, and the factors affecting each of these systems over the course of a lifetime are very complicated. We need to stop seeing healthcare as a one-size-fits-all solution and instead focus on creating more precise diagnoses and individualised treatment programmes in order to better approach an inclusive healthcare system. Biomarkers are used to identify high-risk people and the early stages of sickness, which helps in diagnosis, prevention, and evaluating the effectiveness of therapy [98]. Keep track of ongoing and planned open-source data-sharing initiatives, especially those pertaining to NIH-funded projects (see the required data-sharing policies). Respect government and funding agency mandates on recruitment of individuals from diverse origins and races, use of sex as a biological variable, and dissemination of unfavourable results to the scientific community in order to guarantee scientific rigour and reproducibility [99]. Collaborate, talk, and use data harmonization techniques with biostatisticians, bioethicists, and AI experts from different research, institutions, and neurodegenerative illness domains. Integrate real-world data into clinical trial programmes; to guarantee health equity. This may include consolidating several initiatives and patient cohorts under one roof [100].

## 7 PRIVACY AND SECURITY ISSUES WITH PRECISION MEDICAL DATA AND THEIR SOCIAL, LEGAL, AND ETHICAL CONSEQUENCES

An assessment of ontology-based precision medicine, a novel healthcare technique, must address ethical, legal, and social (ELSI) problems. Specific norms, moral standards, and fair information principles must compensate for the lack of regulations [101]. However, their effectiveness is restricted in the (mostly) unregulated domains of future health that need context-setting. The Healthcare Information Professionals Code of Ethics, created by Kluge under the guidance of IMIA, has been adopted. It covers the following subjects, among others: autonomy and human rights, exceptions to the rule that rights cannot be realized, exceptions to relevant distinctions between rights and their actualization (practice), a duty to do the best possible action, and guarantee of the range of priorities (logical, natural, voluntary) [102]. The author proposed the following: transparency and openness, limitations on the gathering, sharing, and use of data, security, and access control. In light of the environment's rising complexity and openness, as well as the growing absence of restrictions concerning processes, players, roles, and other factors, the following is required: regulating many policy domains, executing dynamic policy management, and overseeing the adjustment of roles and duties. Big data is made up of various media types and structures, which need to be linked, matched, cleaned, and transformed across systems [103]. The importance of ownership, fairness, context, permission and choice, reasonableness, content, access control, and responsibility serve as a pertinent guideline for using big data and analytics in a way that is both legally and morally correct in order to address this challenge [104].

### 7.1 Access and Societal Impacts to Precision Medicine Therapies

Precision medicine therapies represent the ideal future of medicine. However, critics argue against them, citing the current inability to bridge the gap between vision and reality. Traditional healthcare decision-making focuses on determining which programs to fund within limited budgets. There is particular concern about the applicability and effectiveness of individualized precision medicine techniques in public health. The Melinda Gates Foundation and Bill indicate the growing interest in this strategy throughout the world [105]. Furthermore, in 2018, two distinct international events on PPH were held by the Rockefeller Foundation and the Western Australian Department of Health.

## 8 CHALLENGES AND FUTURE DIRECTIONS

Ontology-based precision medicine in neurodegenerative diseases offers both challenges and opportunities. The main obstacle to the development of effective disease-modifying drugs is our understanding of the variety of these illnesses and whether or not they represent separate states. Future directions include developing AI-driven medical decision support systems, integrating AI with various diagnostic modalities, and thoroughly evaluating AI models in clinical settings. Future possibilities for ontology-based precision medicine in neurodegenerative illnesses include merging many advanced methods. The effective use of precision medicine concepts in disease-modifying therapy depends on overcoming obstacles in comprehending the complexity and heterogeneity of neurodegenerative disorders. To create disease-modifying therapies and biomarkers specific to each patient, a biological model of neurodegeneration must replace the conventional clinicopathologic model. Similar to advances in cancer research, the move towards a holistic systems-based approach to precision medicine shows promise for revolu-tionising the identification, management, and prevention of neurodegenerative illnesses. In the field of neurode-generative disorders, precision medicine can lead to tailored therapies that improve patient outcomes by embracing these developments and addressing disease heterogeneity. This discovery motivated more clinical research to find out if patients with advanced or resistant cancer might benefit from pathway-based molecular profiling (MP)-guided treatment.

## CONCLUSION

Precision medicine is a new field that aims to treat neurodegenerative disorders by tailoring treatments to the requirements of individual patients. However, because diseases are diverse and current techniques of classifying illnesses are insufficient, precision medicine faces challenges. To overcome these obstacles, academic institutions and medical centres must form cooperation networks in order to execute precision medicine strategies that are effective. PrimeKG (a multimodal knowledge graph) integrates several resources to enhance knowledge of disease biology and the relationship between genetic factors and clinical phenotypes, hence supporting customised diagnostic strategies and targeted therapeutics in precision medicine investigations. The advent of technology has accelerated the discovery of genomes by providing tailored treatments based on distinct genetic origins of disease. To overcome these challenges, a biological model is proposed in lieu of the traditional clinicopathologic paradigm, emphasising the need for accurate diagnosis and therapy based on causal molecular pathways. Ontologies redefine and categorise diseases according to their molecular causes. It is crucial to concentrate on determining molecular, pathological, and biomarker-based disease progression in order to guide the creation of innovative therapeutics for diseases in order to enhance precision medicine methods. Through the modulation of medication response and efficacy through miRNA-mediated processes, pharmaco-epigenomics, and especially the study of microRNAs, provides intriguing insights into personalised therapies. Moreover, the development of therapies and patient stratification for clinical trials might be guided by the molecular categorization of neurodegenerative illnesses based on thorough morphological examination of pathologically changed proteins. A potential therapeutic approach to address neurotrophin resistance in age-related neurodegenerative disorders is to target neurotrophin receptor signalling with small-molecule ligands. This highlights the significance of developing precision medicines that target these systems for future healthcare challenges.

## Figures and Tables

**Fig. (1):**
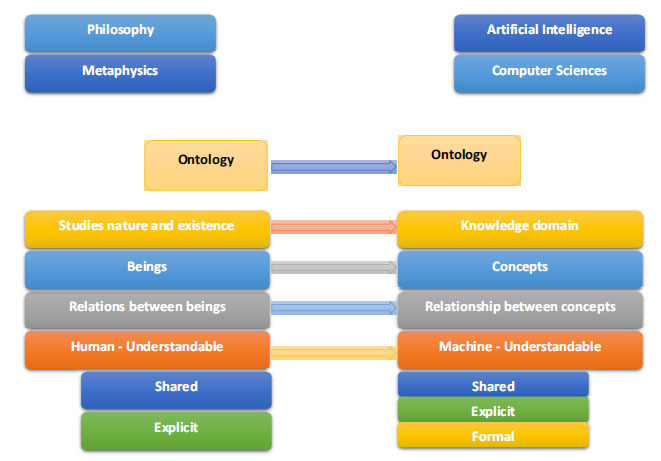
Meaning of ontology in two different contexts.

**Table 1:** Analysis of the clinical, neuropathological, and pharmacological dimensions of Huntington's disease (HD), Parkinson's disease (PD), and Alzheimer's disease (AD).

-	**HD**	**PD**	**AD**	**References**
Etiology	• In all cases, genetic etiology • ITI 5 gene HD • Mutations are excessive CAG	• Most cases are sporadic (environmental insults) • Genetic cases (a- synuclein, parkin, PINK1, LRRK2) described	• Most cases are sporadic • Genetic cases (APP, PS1, PS2) and risk genes (APOE) described	[38]
Neuro-pathology	• Intranuclear inclusions of mutant huntingtin • Degeneration of striatal projecting neurons • Cortical degeneration	• Cytoplasmatic protein inclusions (Lewy bodies) • Dopaminergic denervation of the striatum • Death of nigrostriatal neurons	• Senile plaques (β-amyloid aggregates) • Neurofibrillary tangles (hyperphosphorylated tau protein) • Neuronal losses in cortical &subcortical	[39]
Symptoms	• Chorea • Cognitive dysfunction • Psychiatric symptoms	• Bradykinesia, postural disturbances, rigidity, resting tremor, non-motor symptoms	• Progressive deterioration of cognition and memory, leading to dementia	[40]
Therapies	• Tetrabenazine (VMAT inhibitor) • Autophagy enhancers, inhibitors of histone deacetylases	• Dopaminegic replacement (levodopa, MAO inhibitors, dopamine agonists) • Surgical procedures • Neuroprotective agents and cell	• Acetylcholinesterase inhibitors • NMDA antagonists • Inhibitors of β- and γ- secretases, immunotherapy and inhibitors of tau phosphorylation under investigation	[41]

**Table 2:** Biomarker list, which is used in neurodegenerative diseases.

**Biomarker**	**Description**	**Depiction**	**Diagnostic Efficiency**	**References**
Amyloid-beta (Aβ)	Peptides formed from the cleavage of amyloid precursor protein, accumulating to form plaques in the brain	Detected *via* PET scans and cerebrospinal fluid (CSF) analysis	High specificity for Alzheimer's; Aβ42/40 ratio in CSF is a strong indicator of disease progression	[55]
Tau Protein	Microtubule-associated protein that forms neurofibrillary tangles when hyperphosphorylated	Measured through CSF analysis and tau PET imaging	Alzheimer's pathology and neuronal damage are indicated by elevated levels of total and phosphorylated tau in CSF	[56]
Neurofilament Light (NFL)	A structural protein of neurons is released into CSF and blood following neuronal damage	Quantified in CSF and plasma using immunoassays	High levels in CSF and blood correlate with neurodegeneration severity and disease progression	[57]
Phosphorylated Tau (p-tau)	Specific forms of tau protein are linked to tau tangles and neuronal damage	Assessed using CSF analysis and tau PET imaging	Increased p-tau levels are highly specific to Alzheimer's, aiding in differential diagnosis from other dementias	[58]
Apolipoprotein E (APOE) ε4	a genetic variation linked to a higher incidence of Alzheimer's	Identified through genetic testing	The presence of the APOE ε4 allele increases risk but is not definitive; useful in risk stratification and early intervention strategies	[59]
Neurogranin	A postsynaptic protein involved in synaptic plasticity is released into CSF during synaptic degeneration	Measured *via* CSF analysis	Elevated levels in CSF are indicative of synaptic dysfunction and correlate with cognitive decline in Alzheimer's patients	[60]
BACE1	the enzyme that converts the amyloid precursor protein into amyloid-beta	Assayed through CSF analysis	Higher levels of amyloid-beta formation and the advancement of Alzheimer's disease are associated with increased BACE1 activity in CSF	[61]
Clusterin (CLU)	A protein involved in amyloid-beta clearance and neuroinflammation	Detected in plasma and CSF *via* immunoassays	Elevated levels in plasma and CSF are associated with Alzheimer's, reflecting ongoing neuroinflammatory processes	[62]
YKL-40	A glycoprotein associated with neuroinflammation and astrocyte activation	Measured in CSF using immunoassays	Increased levels of CSF correlate with neuroinflammation and disease progression in Alzheimer's patients	[63]
MicroRNA (miRNA)	Small non-coding RNAs regulating gene expression, with altered profiles in neurodegenerative diseases	Analyzed through blood or CSF samples using qPCR or sequencing	Specific miRNA profiles can distinguish Alzheimer's from other dementias and may reflect disease stage and response to treatment	[64]
TREM2	Microglial activation and the reaction to amyloid plaques are mediated by a trigger receptor that is expressed on myeloid cells 2	Measured in CSF or blood	Increased risk and development of Alzheimer's disease are linked to elevated CSF levels, which are indicative of microglial activation	[65]
GFAP	A sign of neuroinflammation and astrocyte activation is glial fibrillary acidic protein	Detected in CSF or blood	Elevated levels indicate astrocytic response to neurodegeneration, aiding in disease diagnosis and monitoring	[66]
Neurotrophic factor derived from the brain (BDNF)	Neurotrophins are important for the survival, plasticity, and cognitive processes of neurons	Measured in CSF and blood	Lower values are linked to Alzheimer's disease-related neurodegeneration and cognitive decline	[67]

**Table 3:** Examples of conditions in which precision medicine has been used.

**Medical Field**	**Disease**	**Biomarker**	**Intervention**
Cancer Imatinib4 Lung cancer	Chronic myeloid leukemia	Breakpoint cluster region -Abelson Echinoderm microtubule-associated protein-like 4 anaplastic lymphoma kinase	Crizotinib
Hematology	Thrombosis	Factor V Leiden	Avoid prothrombotic drugs
Infectious disease	HIV/AIDS	The cluster of differentiation 4 + T cells, HIV viral load	Highly active antiretroviral therapy
Cardiovascular disease	Coronary artery disease	CYP2C19	Clopidogrel
Pulmonary disease	Cystic fibrosis	Glycine to Aspartate change in nucleotide 1784 in exon 11	Ivacaftor
Renal disease	Transplant rejection	Urinary gene signature	Antirejection drugs
Hepatology	Hepatitis C	Hepatitis C viral load	Direct-acting antiviral agents
Endocrine disease	Multiple endocrine neoplasia type 2	Rearranged during Transfection	Prophylactic thyroidectomy
Metabolic disease	Hyperlipidemia	Low-density lipoprotein cholesterol	Statins
Neurology	Autoimmune encephalitis	C-X-C motif chemokine ligand 13	Immunotherapy
Ophthalmology	Leber’s congenital amaurosis	Retinal pigment epithelium-specific 65 kDa protein	Gene therapy

**Note**: Ref. [68, 70, 71, 89].

**Table 4:** List of *in silico* tools for NDs.

**Tool**	**Description**	**Use in NDs**
BioCyc Database Collection	Include hundreds of microorganisms, 17832 Pathway/Genome Databases (PGDBs) for model eukaryotes, and analysis tools	Examination of numerous NDs' paths
Reactome	A manually maintained, open-access route database that offers basic tools for studying pathways and genomes, modeling, visualising, and interpreting	Examination of the gene ontologies and pathways of NDs
Cytoscape	A free and open-source application for complicated network analysis and visualization	Visualization and analysis of complicated gene and PPI networks in NDs
DAVID	This functional analysis enrichment tool offers tabular annotation	Examine the functional annotation and gene ontology of ND genes.
MATLAB	A tool for determining centrality and researching topology	For assessing centrality and characterizing ND networks
ClueGO	Examining biological enrichment pathways using a Cytoscape add-on	ND pathway analysis utilising the terminology from their gene ontologies
Pathway Linker	A Cytoscape tool that links proteins to signal channels	Sorting, showing, and connecting ND proteins to signalling pathways
BLAST	An NCBI tool for matching biological sequences	Comparing the NDs' gene and protein sequences
FatiGo	An online tool for generating ideas for a collection of genes' gene ontology	Finding the ND genes' gene ontology concepts in connection to other genes
Gephi	An open-source network visualization and analysis tool	ND network building, analysis, and visualisation
Cytohubba	It’s a java Cytoscape plug-in for several topological analyses	This Java Cytoscape plug-in is used for a number of topological investigations.
CluePedia	Another tool for clustering networks and analysis of enrichment pathways	An additional tool to integrate networks and enrichment pathway research
Binom	A plug-in of Cytoscape that enhances the manipulation of biological networks	An add-on for Cytoscape that makes biological network administration simpler
CentiScaPe	A plug-in of Cytoscape that helps in topological analysis, such as centrality measurements	An extension for Cytoscape that facilitates topological analysis, including centrality calculations
MetaCore	Commercially available tool for functional analysis of experimental data	A tool for functional analysis of experimental data that is commercially available
NetworkAnalyzer	Multi-functional tool in Cytoscape for analyzing topological parameters	A multipurpose tool in Cytoscape for investigating topological factors
BisoGenet	Another Cytoscape plug-in for gene network construction and topological analysis	An additional Cytoscape add-on for topological analysis and gene network building

**Note**: Ref. [70-73].

## LIST OF ABBREVIATIONS

ABL: Abelson
AD: Alzheimer's Disease
AIDS: Acquired Immunodeficiency Syndrome
ALK: Anaplastic Lymphoma Kinase
AMD: Age-related Macular Degeneration
APP: Advanced Practice Provider
Aβ: Amyloid-beta
BACE1: Beta-secretase 1
BAO: Basilar Artery Occlusion
BCR: Breakpoint Cluster Region
BDNF: Neurotrophic Factors Derived from the Brain
CAG: Coronary Angiogram
CD4: Cluster of Differentiation 4
CLU: Clusterin
CSF: Cerebrospinal Fluid
CXCL13: C-X-C Motif Chemokine Ligand 13
CYP: Cytochrome P450
DTO: District Tuberculosis Officer
EML4: Echinoderm Microtubule-associated Protein-like 4
G551D: Glycine to Aspartate Change in Nucleotide 1784 in Exon 11
GFAP: Glial Fibrillary Acidic Protein
GO: Gene Ontology
HD: Huntington's Disease
HIV: Human Immunodeficiency Virus
ICD10: International Classification of Diseases, Tenth Revision
IMIA: International Medical Informatics Association
LDL: Low-density Lipoprotein
LIFEo: Last In, First Out
LRRK2: Leucine-rich Repeat Kinase 2
MAO: Monoamine Oxidase
mHtt: Mutant Huntingtin
MPEG: Moving Picture Expert Group
NCI: National Cancer Institute
NDDO: Neurodegenerative Disease Data Ontology
NDDs: Neurodegenerative Diseases
NDF-RT: National Drug File Reference Terminology
NDs: Neurodevelopmental Disorders
NFL: Neurofilament Light
NIH: National Institutes of Health
OntoDM-core: Ontology of Core Data Mining Entities
OntoDT: Ontology of Data Types
PD: Parkinson's Disease
PET: = Positron Emission Tomography
PharmGKB: Pharmacogenetics Knowledge Base
PINK1: PTEN Induced Kinase 1
PM: Precision Medicine
poly Q: Poly-glutamine
PPH: Postpartum Hemorrhage
PrimeKG: Precision Medicine Knowledge Graph
PS1: Presenilin 1
PS2: Presenilin 2
RET: Rearranged During Transfection
RPE65: Retinal Pigment Epithelium 65 kDa Protein
RPE65: Retinal Pigment Epithelium-specific 65 kDa Protein
TCGA: The Cancer Genome Atlas
UMLS: Unified Medical Language System
WHO: World Health Organisation
YKL-40: Chitinase-3-like protein 1
